# Perceptions and experiences of intravenous iron treatment for anaemia in pregnancy in Malawi: a formative qualitative study

**DOI:** 10.12688/gatesopenres.13631.1

**Published:** 2022-05-17

**Authors:** Lucinda Manda-Taylor, Macdonald Kufankomwe, Gertrude Chatha, Effie Chipeta, Elisabeth Mamani-Mategula, Martin N. Mwangi, Magaret Kelaher, Khic-Houy Prang, Ricardo Ataíde, Sant-Rayn Pasricha, Kamija Samuel Phiri

**Affiliations:** 1 School of Global and Public Health, Kamuzu University of Health Sciencies, Private Bag 360, Blantyre, 3, Malawi; 2 Centre for Health Policy, Melbourne School of Population and Global Health, The University of Melbourne,, Melbourne, Level 4, 207 Bouverie Street, Victoria 3010, Australia; 3 Population Health and Immunity/Infection and Immunity Divisions, The Walter and Eliza Hall Institute of Medical, Research, 1G, Royal Parade, Parkville, Melbourne, VIC 3052, Australia

**Keywords:** Anaemia in pregnancy, intravenous iron infusion, maternal and child health, Malawi

## Abstract

**Background**: The study objective was to explore opinions, identify experiences, and describe perspectives on the acceptability of intravenous (IV) iron to treat anaemia in pregnancy and identify potential barriers and facilitators of introducing IV iron in the Malawian healthcare system.

**Methods**: We conducted 15 in-depth interviews and two focus group discussions with pregnant women, and seven in-depth interviews with health workers at a community-based health centre in Blantyre and a tertiary hospital in Zomba.

**Results**: Most women who used IV iron treatment during the second trimester of pregnancy reported feeling better and stronger after receiving the intervention. Women perceived that IV iron treatment worked faster and increased their haemoglobin count. However, cultural beliefs that IV iron treatment will cause miscarriage and the perception that study procedures involved Satanism and vampirism practices were barriers to acceptability. Health workers found IV iron treatment easy to administer because it is a single-dose treatment, simultaneously reducing the burden for pregnant women taking daily oral iron tablets. However, health workers expressed concerns about the costs and the need to train health workers before the large-scale implementation and integration of IV iron treatment into Malawi’s routine care.

**Conclusions**: Despite the perceived concerns and challenges experienced in participating in the first IV iron infusion trial in Malawi, participants’ reflections suggest that IV iron infusion is acceptable for treating iron-deficiency anaemia in pregnancy. Participant advocate groups can offer a peer-to-peer education approach to sensitize and engage community members on the benefits of treatment and dispel concerns when the country contemplates integrating IV iron infusion for treating anaemia in pregnancy in Malawi.

## Abbreviations

IDA: Iron Deficiency Anaemia

COMREC: College of Medicine Research Ethics Committee

DHO: District Health Office

FCM: Ferric carboxymaltose

Hb: Hemoglobin

IV iron Intravenous iron

LMICs Low-middle income countries

MDHS: Malawi Demographic Health Survey

REVAMP: Randomized controlled trial of the Effect of intraVenous iron on Anaemia in Malawian Pregnant Women

## Introduction

Iron deficiency is the most common form of anaemia, with the current estimate that 1.74 billion people are affected ^1^. There are numerous risk factors for anaemia, including age, gender, geography and health status. Gender and age present the most significant risk factors for iron deficiency anaemia (IDA), with women and children having a higher likelihood of developing anaemia. Anaemia in pregnancy remains a critical global health problem, affecting 46% of pregnant women in Africa and 49% in Asia ^2^. In addition, anaemia is a significant contributory factor to birth-related complications such as more substantial blood loss at childbirth and post-partum ^3^, pre-term births, and low birth weight babies.

## Anaemia in Malawi

Anaemia in pregnancy is common in Malawi ^4^. A haemoglobin level below 11.0g/dl confirms anaemia in pregnancy ^5^. The Malawi Demographic Health Survey (MDHS) 2015–2016 reports prevalence rates that indicate that 33% of women are anaemic, 25% are classified as mildly anaemic, 7% as moderately anaemic and 1% as severely anaemic for all anaemia ^5^. Anaemia also varies by maternity status. The MDHS reports that 45% of pregnant women are anaemic compared with 30% that are breastfeeding. Interestingly, women living in urban areas are slightly more likely to be anaemic (36%) than those living in rural areas ^5^.

In Malawi, iron, but not folate, supplementation is part of routine antenatal care (ANC) ^5^. However, the MDHS 2-15-2016 indicated that only one-third of women took iron supplements for 90 days or more, as recommended during pregnancy, while 11% did not take any iron supplements ^5^. Munashinge and van den Broek (2006) suggest that women dislike the tablets because of the smell or taste, resulting in poor adherence to oral iron supplementation ^4^. Nevertheless, the standard treatment for anaemia in pregnancy in Malawi, as in many other low-middle income countries (LMICs), is oral iron tablets due to the low cost, good safety profile, and administration ease.

Ferric carboxymaltose (FCM) is a parenteral iron product and used for rapid and high dose replenishment of depleted iron stores ^6^. FCM contains an iron complex with a ferric hydroxide core that is stabilized by a carbohydrate shell. Its composition allows for the administration of large doses (15mg/kg; maximum of 1000mg/infusion) to replenish the depleted iron stores in the body ^6^. One dose can provide a large amount of iron and has a very short infusion time ^7^. Modern IV iron products such as FCM have become available in developed countries like Australia, Europe, and the United States. IV iron is currently marketed in over 50 countries and recently has become available in Brazil ^8^.

Previous and ongoing studies show FCM to be safe and effective in treating iron-deficiency anaemia. In addition, several randomized Phase III, open-label, controlled, multicenter trials that used oral iron as a comparator have shown FCM to have better efficacy in improving Hb levels in the second and third trimester ^8– 10^. Thus, FCM represents an important therapeutic modality that offers significant clinical benefit and reduces morbidity and mortality from many pathological conditions associated with iron deficiency ^8^. Unfortunately, however, IV iron products like FCM are not yet part of routine treatment for anaemia in pregnancy in many low-income, low-resourced settings like Malawi. Having a really good approach to tackling anaemia in pregnancy may have a positive knock-on effect in reducing maternal mortality and complications associated with childbirth in sub-Saharan Africa.

To address this gap in knowledge, a seminal, large randomized controlled trial of the effect of intravenous iron on anaemia in Malawian pregnant women (REVAMP) is being conducted. The study aims to assess whether, in Malawi, the treatment of moderate to severe antenatal anaemia with IV iron improves critical maternal (including anaemia and wellbeing) and neonatal (including birth weight, gestation duration) outcomes and is safe (infection, hypophosphatemia) compared to oral iron (delivered via the standard of care mechanisms) ^11^. However, the main results of this study are yet to be published, as the trial is ongoing. Meanwhile, a formative qualitative study aimed to explore the opinions, experiences and describe perspectives on the acceptability of IV iron to treat anaemia in pregnancy was conceptualized and subsequently conducted. This is the focus of this manuscript.

## Methods

The article draws on qualitative data collected in Malawi's two districts, Blantyre and Zomba. The study objective was to explore opinions, identify experiences, and describe perspectives on the acceptability and feasibility of administering IV iron to treat anaemia in pregnancy in Malawi.

### Data collection procedures

We used a semi-structured interview guide for the in-depth interviews (IDIs) and focus group discussions (FGDs) (see *Extended data* ^12^). The IDI and FGD questions administered to pregnant women related to concepts, procedures, experiences and health outcomes. Specifically, we asked the women their experiences when they visited the ANC, including their experiences with the medication, treatment, and care they received. We also wanted to know their views on the acceptability of IV iron and what process they used to decide to join the study. The IDI questions for health workers focused on perceptions about the benefits and challenges of administering IV iron treatment and the enabling and constraining factors to integrate IV iron treatment within the ANC continuum of care model for pregnant women diagnosed with moderate or severe anaemia. The tools were piloted by a researcher and health worker to check for clarity, relevance, comprehensiveness, and question flow. Unfortunately, we could not pilot our interview guides on pregnant women because of their availability challenges during our interview piloting phase. Despite that, we felt confident that the tools would work because we piloted them on a female researcher with experience with ANC care. Questions that we identified as ambiguous and not relevant to answering the main study objectives were amended or removed.

Owing to the exploratory nature of this research, sample size was not formally calculated. However, we aimed to interview 12-15 pregnant women at each participating site and conduct at least 2 Focus Group Discussions (FGDs) with pregnant women and 2-10 IDIs with health care workers. For the FGDs, our minimum sample size was between 6-12 participants per FGD.

Participants were purposively selected using a criterion-sampling strategy to identify and choose the pregnant women who had received IV iron treatment and health workers who had administered the intervention (as part of the REVAMP trial). The sampling strategy was chosen because we wanted to interview individuals who had experience receiving and distributing the intervention. They would possess first-hand knowledge of IV iron treatment and provide detailed information, making them information-rich participants. We identified the eligible women who had participated at both REVAMP trial sites in Blantyre and Zomba. The trial site coordinators provided us with the participant ID and contact numbers. Participants were called by telephone and invited to participate in an interview or focus group discussion. Only those who had confirmed availability and expressed a willingness to participate were invited on a mutually agreed date and time.

Data were collected from December 2019 to January 2020 by one Malawian research assistant trained on the protocol and data collection methods and conducted the interviews and focus group discussions. The research assistants were familiar with the local context and spoke the local language (Chichewa).

The IDIs and FGDs were held in private spaces. For instance, the IDIs and FGDs were conducted in private rooms at the two health facilities. We conducted the IDIs and FGDs with the pregnant women in Chichewa (the local language), and audio-recorded (with permission) and later transcribed in Chichewa and then to English. The health worker interviews were all conducted in English. The interviews lasted anywhere between 45 to 60 minutes. The IDIs provided most of the data used in the analysis. Towards the end of the field-work period, the same concepts, ideas and perspectives were re-emerging in the in-depth interviews. After the point where the data material appeared complete, a few more interviews were conducted, affirming the impression that thematic saturation had been reached ^13^. The FGDs did not add any new themes but instead confirmed what had already been expressed in the interviews.

### Data analysis

Data analysis was iterative and ongoing throughout the data collection process. The research assistants [MK, GC] and the principal investigator [LMT] checked the transcripts against the recorded interviews for completeness. Next, the research assistants and the principal investigator coded data separately and agreed upon a final coding scheme for all the data collected. Using qualitative data analysis software, QSR NVivo 12 (RRID: SCR_014802), we performed a thematic comparative analysis to identify key concepts that emerged from the interview data ^14, 15^. This analysis consisted of identifying common ideas in the data, exploring relationships among the data to identify patterns, and grouping the data into thematic categories. Emerging categories were compared to identify higher-level themes that explain participant and health worker perceptions, experiences, and attitudes towards IV iron treatment.

### Ethical considerations

The University of Malawi’s College of Medicine Research and Ethics Committee (COMREC) granted ethical approval for the research (P.06/19/2711) on 8 ^th^ September 2019. All methods were performed in accordance with the Declaration of Helsinki and the COMREC guidelines on Health Research. Also, permission to collect data in the health facilities was obtained from the District Health Office (DHO) in Blantyre and Zomba. Written informed consent was provided in Chichewa or English, per the participant's language preference. Literate participants provided a signature on the consent form, and illiterate participants provided a thumbprint. Participants were informed that confidentiality would be maintained and that no personal details would be divulged. Lastly, participants were told that their involvement in the research was voluntary and that withdrawal was permitted at any time and without personal consequence.

## Results

We conducted 15 in-depth interviews (n=9 in Blantyre (BT) and n=6 in Zomba (ZA) districts) and 2 FGDs (n=6 and n=8) with pregnant women, one in each district (Blantyre and Zomba). The FGDs with pregnant women were additional methods (triangulation) to collect data on the same topic and validate our findings. The majority of the pregnant women interviewed had given birth before, with only 9 out of the 29 women carrying their first pregnancy. We also conducted seven IDIs with health workers (n=2 in BT and n=5 in ZA). All health workers we interviewed were employed under the REVAMP trial. One health worker we interviewed was a medical doctor, while the others were qualified health care specialists with a nursing background. All participants were 18 years and above. Refer to Table 1 and Table 2 below.

**Table 1.** Pregnant women in-depth interviews and focus group discussions, Blantyre and Zomba Districts.

	In-depth interviews	Focus group discussion (n=2)
**Participants N**	15	14
**Age groups (years)**		
*15–19*	3	1
*20–24*	4	4
*25–29*	3	5
*30–34*	0	3
*35–39*	5	1
Parity*		
*0*	5	4
*1*	4	5
*2*	3	3
*3*	0	1
*4*	1	0
*5*	2	1
**District & Health facility**	**No. of** **participants** **per district**	
*Blantyre/ Limbe Health Centre*	15	
*Zomba/ Zomba Central Hospital*	14	

**Table 2.** Health worker in-depth interviews.

	N
**Age groups (years)**	
*25–29*	1
*30–34*	3
*35–39*	1
*40–44*	2
**Education**	
*Medical Degree*	1
*Diploma, Nursing and Midwifery*	5
*Degree, Nurse Management*	1
**Residence**	
*Blantyre*	2
*Zomba*	5
**District & Health facility**	
*Blantyre/ Limbe Health Centre*	2
*Zomba/ Zomba Central Hospital*	5

Our study generated three main themes under the topics on perceptions and experiences of IV iron treatment. These themes coalesce around the experiences with receiving and administering IV infusion, concerns with IV infusion and taking blood and factors that would enable or hinder IV iron’s acceptability for treating anaemia in pregnancy. Of note here is that all of the pregnant women we interviewed presented a general knowledge and understanding of anaemia in pregnancy. In addition, they were able to identify the signs and symptoms of anaemia.

### Experiences and consequences with IV iron infusion

Women reported experiencing fear and apprehension about receiving IV iron treatment. Most fears that the women described were about the procedure of receiving an injection. Tolerability was, however, one specific concern that a woman expressed. “I was worried about whether my body will tolerate it and whether it will yield good results” (IDI-PW-02-BT). Apart from tolerability, some women expressed feeling ill after receiving the intervention. These feelings were described as having shortness of breath, difficulty walking, temporary sight impairment and headache.

A health worker also expressed initial apprehensions with administering the intervention but observed that only a few women reported feeling ill. In addition, the side effects the women experienced were manageable over time.

“I was anxious that maybe there would be some side effects, but after administering the first drug, it was ok. The second and the third time, I saw that it is fine. Even the side effects which were anticipated, such as headache and general body pains, only a few people reported them. For example, out of 20 participants, only one can report experiencing a headache. So in this study, since I started, maybe only one or two have reported having side effects, but we have administered the drug to so many people.” (IDI-HW-27-ZA).

### Concerns with IV iron infusion

Although there was general acceptance of receiving and administering IV iron infusion, studies in communities will always raise suspicion. These suspicions are amplified if the interventions are new. The women we interviewed expressed community concerns about miscarriage, Satanism and vampirism. As one woman revealed, “There are misconceptions that people say concerning studies or about this study, for example, they say it makes one miscarry; hence many people fear to join this study.” (IDI-PW-11-BT). Another woman informed us that her landlord said once that “the blood samples collected from us, you sell them” (FGD-P3-01-ZA).

The health workers we interviewed also reported similar community misconceptions and the challenges they experienced recruiting and retaining participants.

“Because we get the blood samples, and after delivery, we also need some things like placenta tissue, placenta membranes, and umbilical cord blood people now connect this with Satanism. Every time we take a blood sample, people say that ‘I have already heard that you will take my uterus so that I shall not give birth again’. So, it requires us to sit down with them and explain again to them that we do not take all uterus and that they will give birth again.” (IDI-HW-15-BT).
“Most of the clients I have seen withdrawing are from Bangwe [township]. Most of the Bangwe based clients are talking of bloodsuckers. I do not know why it is like this in this community; maybe it is because of bloodsuckers’ issues that are speculating around. So, I believe it is because of that or whatever people just connect.” (IDI-HW-15-BT).

Additional concerns were expressed by the participants related to confusion over the colour of the treatment.

“Upon looking at the colour of the medication as it was red, I was worried. I thought that the medication was harmful, and can harm my child. I said to myself, ‘what will happen to my child? Will it not kill my child’?” (FGD-P6-01-ZA).

In addition to the concerns around drawing blood, other potential barriers to implementing and administering IV iron infusion to treat anaemia in pregnancy were raised. For example, some participants raised issues concerning the cost associated with procuring the treatment and how the treatment is administered. One pregnant woman, in particular, introduced the concern of experiencing pain with the procedure.

“I was feeling pain because they could not see the vein. They had difficulties finding the vein, and when they pierced me, no blood was coming out of it. As a result, they kept trying to find a vein that could give them blood to put the cannula. Luckily the vein was found though they said the veins were tiny, but after they tried so many times. They had to put plaster in places where I was pierced to avoid blood coming out.” (IDI-PW-07-BT).

Health workers echoed the above concern because they experienced a reluctance from participants to be injected with FCM.

“Yes, some people are afraid of being pierced with an injection or even yourself. If we try to pierce you with a syringe needle, you will show some fear. So, some people have that fear, so with the cannula, you want it to get into the vein and some people, even after you have explained and when you are trying to pierce them, they do twitching, so such things are the common challenges.” (IDI-HW-21-ZA).

Another health worker mentioned the concern around the time it takes to administer the intervention as a possible barrier since the infusion of the drug takes about 15 minutes and can cause an adverse reaction.

“Sometimes, during infusion, the drops stop, and a client's hand starts swelling. We remove and search for another vein because if we continue, the client can develop another problem as the drug goes straight in the tissues, not in the vein.” (IDI-HW-11-ZA).

### Conditions supporting the administration of IV iron

There were shared views on the perceived benefits of IV iron infusion between women and health workers. Participants identified advantages related to the single treatment dose and perceived improvements in health and maternal-child health outcomes. In addition, most women reported having started feeling better and getting stronger a few days or up to two weeks of receiving the intervention.

“After three days I felt a change, even I went to the garden, and people began to wonder how come I am looking healthy and well now, and they were saying I was abusing myself because they thought that I am sick, but to my me I was fine.” (FGD-P5-01-ZA).
“After joining the study, I was given IV Iron therapy, and within two weeks, I experienced a change in my body because then I was experiencing dizziness, a problem in seeing as I saw darkness, and my legs were swollen, but all these ended within two weeks” (IDI-PW-05-BT).

Furthermore, one health worker had “witnessed that those women who have taken IV therapy give birth to children of adequate birth weight and even more than that.” (IDI-HW-27-ZA).

### Strategies to consider when introducing IV iron infusion

To further explore what methods would facilitate the implementation of IV iron treatment in Malawi, the pregnant women interviewed suggested using participant advocates as part of public or community engagement strategies.

“If you want to give this treatment to a broad population, there is a need that you should use the people who have used the therapy when giving out messages relating to the treatment in terms of its effectiveness. These people should be telling their fellow women the importance of this treatment as they have used it before. Explaining their experience with the treatment to their fellow pregnant women can help remove negative altitudes that I had of which other women also have towards this treatment” (IDI-PW-02-BT).

Another woman echoed the above view who suggested informing women about this treatment at places in the communities where they commonly meet. “We can even use places where we women get water because this is where women meet in large numbers. So bring this issue at this place, I think someone can be interested and learn something” (FGD-P5-01-ZA).

One health worker echoed this strategy and recommended using patient participants as health advocates to dispel some of the treatment’s confusion and myths.

“On that one, I can say, it needs sensitization for the people to understand. For example, people from religions that do not receive the blood do not understand that what we are giving is a medication because FCM, when diluted, appears like blood, so it needs sensitization that it is the same medication, not blood. But, still, when it is diluted, it looks like blood.” (IDI-HW-21-ZA).

Other strategies proposed included radio programs to inform the public of the benefits of receiving IV iron treatment for pregnant women with anaemia.

“You can also use the radios to spread the news about this medication. Then, I think it will be easier and fast to reach more people. People should also be told about this medication in hospitals, and posters should also be used. Then, people can start believing and gaining the courage to participate” (FGD-P3-01-ZA).

Building health workers' capacity through training was a critical strategy to deliver the intervention. Lastly, engaging local and national stakeholders to present the REVAMP trial’s evidence was vital in influencing a policy change.

“If all stakeholders put their minds together, put their interests together, their hands together, and agree to say this is the way to go as Malawi. We could recommend it to policy-makers that look at this trial; it has achieved, informed policy, and informed science; so why don’t we recommend it?” (IDI-HW-08-BT).

Presenting the REVAMP trial results to health policy-makers will inform all health policy stakeholders and plan the challenges and health system bottlenecks that need addressing.

## Discussion

This is the first exploratory, qualitative study to report on pregnant women’s experiences with receiving IV iron treatment and health workers' experiences with administering IV iron treatment to pregnant women in the REVAMP clinical trial. The pregnant women and health workers we interviewed reported that no serious adverse events were experienced or recorded. In addition, the pregnant women experienced an improved sense of wellbeing, clinically, physiologically and physically, after infusion. The pregnant women’s accounts of their experiences are supported by clinical trial studies conducted elsewhere, demonstrating the tolerability, safety and efficacy of FCM infusion and the quality of life improvements in maternal and neonatal health ^16, 17^. However, the study also highlighted barriers to implementing IV iron infusion in Malawi. The barriers fell into two main groups: hindrances due to community acceptance and health system factors.

### Acceptability of IV iron for pregnant women in Malawi

This is the first study in Malawi to describe participants’ perceptions and experiences of intravenous iron treatment for anaemia in pregnancy. This formative study’s findings reveal the preference for IV iron infusion as a single dose therapeutic. IV iron was tolerable and, to this end, acceptable to women who received the treatment and experienced the benefits. However, some significant barriers or conditions would affect health system implementation and community acceptance and affect uptake among women who might benefit from the treatment. 

### Conditions affecting the acceptability of IV iron infusion

The health workers in the trial expressed their challenges in recruiting and retaining participants, mainly because of superstitious narratives and perceptions about the research. Participants’ superstitious perceptions of biomedical research in Africa are deep-rooted and widespread, and Malawi is no exception ^18, 19^. These views stem from the colonial era when White colonial health officials came in cars and vans and collected blood from Africans ^20^. While beliefs and fears of witchcraft and “bloodsuckers” are still prevalent today, these present-day bloodsuckers – who are not of the Dracula variety – are viewed as strangers in the community ^20^. Stories of strangers that include Malawian, non-Malawian or White health researchers who move around in villages recruiting research participants, drawing or collecting biospecimens are pervasive in literature. Health researchers and doctors have been likened to vampires, but instead of having fangs, they use needles and unspecified technology to steal people’s blood ^20^. As Sharra observes, “the shift from traditional medicine to advanced health management systems, which involves the collection of blood samples to detect infections and treat diseases, creates and reinforces a suspicion” ^21^. Similar superstitions and concerns emerged in this study with collecting biospecimens (even though specimens were only collected during the trial and will not be collected in routine care). The worries about blood taking exist because blood represents life or death ^22^—the taking of blood links to women’s concerns about miscarriage and the selling of blood ^23^.

These perceptions presented a challenge to the recruitment and retainment of study participants and challenged the notion of acceptability. Geissler and Pool caution medical researchers to not reject or ignore such perceptions because they sometimes impede recruitment to research, affect adherence to interventions and even threaten the whole continuation of research ^23^. Furthermore, they suggest that perceptions and concerns such as these contain local interpretations of medical research ethics ^23^, especially regarding a lack of proper community engagement in medical research and public health interventions.

### Health system implementation barriers

In this study, FCM was provided free of charge to the participants in the study. Nevertheless, health workers in the trial mentioned the cost of treatment as a barrier. Published literature also suggests that the cost of screening for IDA in limited-resource settings is prohibitive ^24^. In Malawi’s public health facilities, health services have been provided freely by the government since it gained independence from colonial rule in 1964 ^25^. The exemption of user fees is to expand access to health care ^26^. Through the Ministry of Health, Malawi's government is responsible for providing cost-effective interventions targeting a limited number of diseases and conditions that rank highly in disease burden ^26^. Literature suggests that treatment with FCM versus oral iron tablets is cost-effective despite the higher acquisition cost ^27^. However, this study did not evaluate the cost-effectiveness of introducing this intervention. Nevertheless, we can surmise that the single dose of FCM required to replenish iron stores in pregnant women with anaemia would provide a cost-efficient way to reduce maternal and neonatal mortality morbidity.

There is a lack of literature on implementing evidence-based practice in rural and remote settings ^28^. Health workers in the REVAMP trial suggested that another significant barrier to overcome upscaling the intervention is to provide training to frontline health workers (for example, midwives, doctors, pharmacists, laboratory technicians) to detect and treat anaemia in pregnancy with FCM. The development of a protocol to guide detection and treatment by health workers providing antenatal care (ANC) will be essential to guide clinical decision making. The REVAMP trial protocol and the protocol standard operating procedures (SOPs) for detecting and treating anaemia in pregnancy would be a useful starting point ^24^.

Overall, our study’s health workers saw the implementation of IV iron infusion in pregnancy as highly feasible. However, there were considerable concerns about health system issues that would need to be addressed before the full-scale implementation of FCM to treat anaemia in pregnancy in low-resource settings like Malawi.

### Conditions supporting the acceptability of IV iron infusion

Some pregnant women we interviewed suggested using participant advocates to help ease the introduction and implementation of IV iron treatment for pregnant women in Malawi. Beyond the exercise of community engagement, we argue that researchers and research teams must promote collaborative partnerships in research for the research to be perceived to have social value and scientific validity. The engagement of communities in research will help address the misunderstanding surrounding the colour of the treatment and learning about the practical challenges of introducing a new intervention. Greiten *et al*. propose that these concerns present an opportunity for the medical staff within international research teams to develop culturally sensitive communication engagement strategies that help increase the information provided to communities through some form of community participation (e.g., inviting patients to visit the laboratories), and sensitization or even avoiding the colour red in symbols and products related to the intervention ^22^. When research is culturally sensitive and genuinely focused on community benefits, it meets the criteria for ethical research in low-resource settings ^29^.

## Strengths and limitations of the study

Our study has some limitations, the main one being that the participants we interviewed were those directly involved with or who participated in the study. We did not interview other health providers or health professionals who, in the future, would be involved in the implementation of IV iron infusion in routine antenatal care should IV iron become the standard treatment of care for pregnancy-related anaemia. Also, because we only collected the participant’s perceptions and experiences, we may have ignored other people’s views. Broader opinions from family members, community members, and traditional and religious leaders would also be necessary for understanding the acceptability of IV iron infusion beyond immediate participant views. Nonetheless, our study provides insights from individuals who had first-hand experience. These opinions and recommendations may accurately convey their experiences and help decrease the risk of misinformation on IV iron infusion for treating anaemia in pregnancy.

## Recommendations

Health research is essential for patients because it helps discover new ways to diagnose and treat disease by developing efficacious therapies to improve life quality ^30^. We recommend that active engagement with communities be fundamental to the broader acceptance, uptake, and adoption of IV iron infusion to treat anaemia in pregnancy in Malawi. Therefore, we propose establishing patient advocate groups (PAGs) comprised of previous study participants to educate patients and others in the community about IV iron infusion. The use of PAGs would involve recruiting a small cohort of volunteers who had participated in the research. These volunteers familiar with the research procedures and the potential clinical outcomes a participant may experience would provide peer support to participants enrolled in a trial and receive the health intervention. These volunteers would be provided compensation for travel and a small stipend to reflect their time commitment as research advocates ^31^. In this way, PAGs will help build a bridge of understanding by serving as partners with researchers and allies with patients ^32^.

Moreover, the involvement of PAGs in research to treat pregnant women with iron deficiency anaemia would complement the role that Community Advisory Groups (CAGs) play in engendering acceptable, ethical practices in health research in resource-constrained settings ^33^. A further study evaluating the cost-effectiveness and cost-benefits of introducing FCM in the Malawian health care setting would help address this concern. Finally, context-specific research needs to be conducted into how evidence and practice from the REVAMP trial can be implemented in context-specific ways ^28^. 

## Conclusion

Despite the apparent concerns and challenges experienced in participating in the first IV iron infusion trial in Malawi, participants’ reflections suggest that IV iron infusion is acceptable for treating iron-deficiency anaemia in pregnancy. Participant advocate groups can offer a peer-to-peer education approach to sensitize and engage community members on the benefits of treatment and dispel concerns when the country contemplates integrating IV iron infusion for treating anaemia in pregnancy in Malawi.

## Data availability

### Underlying data

Datasets used and analyzed during the current study are not publicly available because consent was not obtained from the participants to make their data public. However, individuals interested can apply for access to the data if they wish to replicate the study, analyse the data or reuse it. One can access the dataset by clicking the link https://doi.org/10.7910/DVN/1CHREM ^12^ and request permission to access the dataset.

### Extended data

Harvard Dataverse: Replication Data for Perceptions and experiences of intravenous iron treatment for anaemia in pregnancy in Malawi: A formative qualitative study. https://doi.org/10.7910/DVN/1CHREM ^12^.

This project contains the following extended data:

- Appendix 1A IDI Guide Pregnant Women.docx
- Appendix 1B FGD Topic Guide.docx
- Appendix 1C IDI Guide Health Workers.docx

Data are available under the terms of the Creative Commons Zero "No rights reserved" data waiver (CC0 1.0 Public domain dedication).
