# Supplementary material for: Perceptions and experiences of intravenous iron treatment for anaemia in pregnancy in Malawi: a formative qualitative study
Source: Gates Open Res. 2024 Mar 8;6:66. Originally published 2022 May 17. [Version 4] doi: 10.12688/gatesopenres.13631.4 (PMC10917769; doi:10.12688/gatesopenres.13631.4)
Supplement: Peer review history

# Reviewer report: Reviewer response for version 4

**DOI:** 10.21956/gatesopenres.16803.r38760

**Published:** 2024-12-14

**Authors:** Tanmay Bagade

**Affiliations:** 1 The University of Newcastle, Callaghan, New South Wales, Australia

The article is well-written and uses a robust methodology. Please add some common causes of anaemia in pregnancy in the context of Malawi and add relevant short critiques in the discussion section. For example, you can comment on the overarching issues of anaemia, such as short birth intervals, malnutrition, recurrent malarial infections, and poor dietary habits, which are some of the causes of anaemia.

Is the work clearly and accurately presented and does it cite the current literature?

Yes

If applicable, is the statistical analysis and its interpretation appropriate?

Yes

Are all the source data underlying the results available to ensure full reproducibility?

Yes

Is the study design appropriate and is the work technically sound?

Partly

Are the conclusions drawn adequately supported by the results?

Yes

Are sufficient details of methods and analysis provided to allow replication by others?

Yes

Reviewer Expertise:

maternal and child health, gender equity, justice health

I confirm that I have read this submission and believe that I have an appropriate level of expertise to confirm that it is of an acceptable scientific standard.

# Reviewer report: Reviewer response for version 4

**DOI:** 10.21956/gatesopenres.16803.r39001

**Published:** 2024-12-11

**Authors:** Sunil Bhandari

**Affiliations:** 1 Nephrology and Transplant Medicine, Hull University Teaching Hospitals NHS Trust and Hull York Medical School (Ringgold ID: 4020), Hull, England, UK

The authors have presented results of a qualitative explorative study of use of IV iron in pregnant women.

 I would normally expect the main paper results on efficacy and safety to be published first before any secondary papers as is normal practice for a trial - I would recommend the authors check this carefully.

 In addition the data is from 2020 so I am surprised that the main paper is still pending.

 The authors have used appropriate methods using semi-structured interviews and focus groups in pregnant women and health workers. Numbers are small but have generated suitable data. 

 The thematic analysis has generated some important findings which will help in increasing the use and acceptance of IV iron in the wider community and some of the barriers. The main emphasise is education to detail the overwhelming benefits to mother and baby.

Is the work clearly and accurately presented and does it cite the current literature?

Yes

If applicable, is the statistical analysis and its interpretation appropriate?

Partly

Are all the source data underlying the results available to ensure full reproducibility?

Yes

Is the study design appropriate and is the work technically sound?

Yes

Are the conclusions drawn adequately supported by the results?

Yes

Are sufficient details of methods and analysis provided to allow replication by others?

Yes

Reviewer Expertise:

Clinical researcher in clinical trials and expert in iron biology and use of Iron in various populations. I have published widely and written the Anaemia guidelines for the UK in patients with chronic kidney disease including pregnant women with kidnew disease. Also delaying kidney progression

I confirm that I have read this submission and believe that I have an appropriate level of expertise to confirm that it is of an acceptable scientific standard, however I have significant reservations, as outlined above.

# Response

**Published:** 2024-12-11

**Authors:** Lucinda Manda-Taylor

**Affiliations:** Kamuzu University of Health Sciences, Malawi

Thank you, Dr Bhandari,

 We appreciate the time you took to review our manuscript. Your comments on the value of our article are positively pleasing. However, allow me to respond to some of your concerns, namely, 

 1. I would normally expect the main paper results on efficacy and safety to be published first before any secondary papers, as is normal practice for a trial. I would recommend the authors check this carefully.

 Response: We need to be informed of a hard and fast rule about when secondary papers should be ready. Qualitative research is used at all trial development and reporting stages, and publishing qualitative study results is also permissible at these stages.

 Given that this was a formative qualitative study using an exploratory design, it was appropriate to share preliminary insights into challenges and opportunities. Clinical trial data analysis tends to happen after the final trial participant completes the follow-up visit. Qualitative research is used at all stages of trial development and reporting. They can be particularly helpful in developing and evaluating complex interventions. Besides, our work was independent of the clinical trial team, with clear objectives for reporting the participants’ concerns and experiences. We wanted to understand whether the intervention could and should be implemented by assessing their acceptability to intervention by users and healthcare professionals. 

 2. The data is from 2020, so I am surprised that the main paper is still pending.

 Response: The results of the main papers and subsequent papers are available. I've included two citations below. 

1. Ferric carboxymaltose versus standard-of-care oral iron to treat second-trimester anaemia in Malawian pregnant women: a randomised controlled trial
  Pasricha, Sant-Rayn et al.
  The Lancet, Volume 401, Issue 10388, 1595 - 1609 (May 2023)
2. Postpartum maternal and infant haematological effects of second-trimester ferric carboxymaltose versus standard-of-care oral iron in Malawi: longitudinal follow-up of a randomised controlled trial
  Mzembe, Glory et al.
  The Lancet Global Health, Volume 12, Issue 12, e2049 - e2058 (December 2024)

We submit our responses for you to review and will be open to further thoughts. 

 Thank you.

 Yours sincerely,

 Lucinda Manda-Taylor

# Reviewer report: Reviewer response for version 3

**DOI:** 10.21956/gatesopenres.16157.r35563

**Published:** 2024-03-06

**Authors:** Stephanie Best

**Affiliations:** 1 University of Melbourne, Melbourne, VIC,, Australia

Thank you for the opportunity to review this manuscript which has been peer reviewed and approved by one colleague and approved with reservations by another. The authors had wide ranging aims for this study to examine pregnant women’s and health care practitioners’ opinions, experiences and perspectives of acceptability of the use of intravenous iron to treat anaemia in pregnancy. In addition, they specifically sought to identify barriers and potential facilitators to the routine use of IV iron into the Malawian healthcare system.

 The introduction provides a useful summary of the known challenges and indicates a gap in the literature. The methods are generally well described including a culturally appropriate approach to recruitment. Results are provided across three main themes that address the multiple aims of the study. Key issues are addressed in the discussion including strengths and limitations.

 Overall, this study provides strong context specific evidence for some of the challenges to implementing the use of IV iron to address anaemia in pregnancy in Malawi. The findings from this work can provide an evidence informed direction for implementation strategies to support future work in this space.

 Please find some minor points for consideration

- Abstract: “ *Women perceived that IV iron treatment worked faster*….” Faster than what?
- Introduction: Fifth paragraph – *Having a really good approach to tacking anaemia*… delete ‘really’ – a little informal.
- Sixth paragraph – *However the main results of this study are yet to be published*…. Revise ‘main’ to clinical.
- Methods: *The article draws on qualitative data collected in*…. add in ‘ *two of* ‘Malawi’s districts…’.
- Data collection second paragraph: clarity about whether focus groups were online or in person.
- Discussion. Health system implementation barriers second paragraph. *There is a lack of literature on implementing evidence-based practice in rural and remote settings*… add in ‘ *in lower- and middle-income countries*’.
- Discussion: Conditions supporting the acceptability of IV iron infusion second paragraph. Revise the sentence ‘ *Elsewhere colleagues and I articulate*…” This paper has several co-authors and it is unclear who ‘I’ is.

Is the work clearly and accurately presented and does it cite the current literature?

Yes

If applicable, is the statistical analysis and its interpretation appropriate?

Not applicable

Are all the source data underlying the results available to ensure full reproducibility?

Partly

Is the study design appropriate and is the work technically sound?

Yes

Are the conclusions drawn adequately supported by the results?

Yes

Are sufficient details of methods and analysis provided to allow replication by others?

Yes

Reviewer Expertise:

Implementation science, research methods

I confirm that I have read this submission and believe that I have an appropriate level of expertise to confirm that it is of an acceptable scientific standard.

# Response

**Published:** 2024-03-06

**Authors:** Lucinda Manda-Taylor

**Affiliations:** Kamuzu University of Health Sciences, Malawi

Dear Associate Prof Best,

 Thank you for reviewing our manuscript and for your helpful suggestions and comments. We have taken care of your questions. 

- Abstract: “ *Women perceived that IV iron treatment worked faster*….” Faster than what?

**Response: **We have added the words “than oral iron tablets”.

  

- Introduction: Fifth paragraph—Having a really good approach to tackling anaemia… delete ‘really’—it’s a little informal.

**Response: **We have deleted the word "really".

- Sixth paragraph—However, this study's main results have *yet to be published*…. Revise ‘main’ to clinical.

**Response: **We have revised "main" and replaced it with "clinical".

- Methods: *The article draws on qualitative data collected in*…. add in ‘ *two of* ‘Malawi’s districts…’.

**Response:** We have added the number "two."

- Data collection: The second paragraph clarifies whether focus groups were online or in person.

**Response:** The focus group discussions were conducted in person and face-to-face. We have made this clarification in the third paragraph.

- Discussion. Health system implementation barriers second paragraph. *There is a lack of literature on implementing evidence-based practice in rural and remote settings*… add in ‘ *in lower- and middle-income countries*’.

**Response: **We have added "in lower-and-middle income countries".

- Discussion: Conditions supporting the acceptability of IV iron infusion second paragraph. Revise the sentence ‘ *Elsewhere colleagues and I articulate*…” This paper has several co-authors, and it is unclear who ‘I’ is.

**Response:** We have removed this statement altogether.

 Once again, thank you very much for taking the time to review and enhance our manuscript.

 Lucinda Manda-Taylor

# Reviewer report: Reviewer response for version 2

**DOI:** 10.21956/gatesopenres.15471.r33726

**Published:** 2023-06-21

**Authors:** Radhika A. G.

**Affiliations:** 1 University College of Medical Sciences and Guru Teg Bahadur Hospital, New Delhi, Delhi, India

1. Could the authors provide the demographic details of the pregnant women who participated in IDI? Was the perception in some way related to the education status?
2. Were the perceptions also related to the experiences witnessed by these women among peers who had received this treatment?
3. Were the multiparas/others asked about treatment for IDA in previous pregnancies/events? Details would be helpful.
4. Were the family members supportive of this treatment?

Is the work clearly and accurately presented and does it cite the current literature?

Yes

If applicable, is the statistical analysis and its interpretation appropriate?

Yes

Are all the source data underlying the results available to ensure full reproducibility?

Yes

Is the study design appropriate and is the work technically sound?

Yes

Are the conclusions drawn adequately supported by the results?

Yes

Are sufficient details of methods and analysis provided to allow replication by others?

Yes

Reviewer Expertise:

Guideline formulation, Systematic Review, Clinical Research

I confirm that I have read this submission and believe that I have an appropriate level of expertise to confirm that it is of an acceptable scientific standard, however I have significant reservations, as outlined above.

# Response

**Published:** 2023-06-24

**Authors:** Lucinda Manda-Taylor

**Affiliations:** Kamuzu University of Health Sciences, Malawi

Dear Dr Radikha

 Thank you very much for your peer review. We appreciate your questions and queries and hope to respond to them satisfactorily.

  

1. Could the authors provide the demographic details of the women who participated in the IDIs? Was the perception in some way related to their education status?

Response: The demographic details of the pregnant women who participated in the IDIs are provided in Table 1. However, what was not collected in the education status of these pregnant women, and therefore no inferences can be drawn from their perception of the intervention.

  

1. Were the perceptions also related to the experiences witnessed by these women among peers who had received this treatment?

Response: Only one woman reported having been influenced by her peers, and we have included this in the results section under strategies to consider when introducing IV iron infusion, and it relates well to the discussion on conditions supporting the acceptability of IV iron infusion specifically as we discuss the usefulness of participant advocates.

 “Yes, I was very happy because I wanted to be given IV iron therapy and experience what my friend told me. I believed that my health would be improved as soon as possible, and indeed, it happened as I thought” (IDI PW 30 ZA).

  

1. Were the multiparas/others asked about IDAs in previous pregnancies/events? Details would be helpful.

Response: Yes, seven women had previous pregnancies, n=4 had one previous pregnancy, and n-3 had multiple previous pregnancies. These women were asked to compare their experiences between oral iron tablets and IV iron infusion. Most reported preferring IV iron infusion because it is a single-dose administration that rapidly restores their iron stores.

 “This is the best and very good pregnancy I have ever had. You know, “In all the pregnancies I have had, I have been having challenges as I was experiencing several illnesses you talk of swollen of the legs, my skin was becoming pale, having frequent dizziness and losing appetite to food and my husband I was always feeling sorry for me every time I became pregnant, but with this pregnancy, since the time I was given IV iron therapy I am a strong woman and healthier than ever” so I am so happy with this drug. I have seen that, in the past two pregnancies, I was falling sick more often, unlike in this pregnancy. Since receiving IV iron therapy, I am not feeling ill anymore. Even my friends are witnessing this by saying, " *i-i-i-I Mimba zina zija ndiye mumavetsa chisoni* *”* (We felt for you during your previous pregnancies because you had to deal with problems). However, now you look good, are not falling sick, and are still doing household chores as if you are not pregnant” (IDI PW 30 ZA).

 We will add previous pregnancy experiences to conditions supporting the administration of IV iron infusion.

  

1. Were family members supportive of this treatment?

Response: All pregnant women were asked who supported their decision-making process. Most women reported that their husbands helped them decide to enter the research study.

 One woman had this to say,

 “My husband assisted me in deciding to participate in this study. When I told him that I was anaemic, he encouraged me to participate in the program because, looking at that I am pregnant, there was no way we could do but participate in the program. Even if some say against us, a new program always has challenges. We could not proceed with participating in this study, but because my husband and I agreed not to listen to people, I am still in this study” (IDI PW-26-ZA)

 “They wanted to see to what extent the medication would help” (P4-FGD-PW-BT).

 One woman informed us that initially, her husband was unsupportive, but because he had attended her ANC visit and received information from the nurses during the recruitment and enrolment period.

 “Yes, I did explain to my husband, but he was also not in support at first as he thought it might not be good. I asked him why he was thinking like that, but he accepted that I should participate in the study because he saw how I had been suffering from anaemia. The good thing was that we were together at the health facility, so the time nurses were explaining to me that we were together, and he understood everything well. When we arrived home, I also asked his views; he said it was okay and that I should go to this health facility “(IDI-PW-02-BT).

 The support from spouses, especially when women receive ANC treatment and care, is crucial in our context and can inform the level of uptake and acceptability of an intervention. Therefore, spouses' roles in decision-making are conditions that support the administration of IV iron infusion.

 We have added these reflections and quotes in the results section and included a reflection on the role of male involvement in decision-making for health.

 We hope that through these additions, we have addressed your primary concerns and strengthened the results.

 Yours sincerely,

 Lucinda Manda-Taylor, PhD.

# Reviewer report: Reviewer response for version 2

**DOI:** 10.21956/gatesopenres.15471.r32755

**Published:** 2022-11-10

**Authors:** Bernd Froessler

**Affiliations:** 1 Department of Anaesthesia, Lyell McEwin Hospital, Adelaide, SA, Australia; 2 Discipline of Acute Care Medicine, University of Adelaide, University of Adelaide, SA, Australia

This reviewer thank the authors for their careful consideration and response to the comments.

Is the work clearly and accurately presented and does it cite the current literature?

Yes

If applicable, is the statistical analysis and its interpretation appropriate?

Yes

Are all the source data underlying the results available to ensure full reproducibility?

Yes

Is the study design appropriate and is the work technically sound?

Yes

Are the conclusions drawn adequately supported by the results?

Yes

Are sufficient details of methods and analysis provided to allow replication by others?

Yes

Reviewer Expertise:

peri-operative and peri-partum patient blood management.

I confirm that I have read this submission and believe that I have an appropriate level of expertise to confirm that it is of an acceptable scientific standard.

# Reviewer report: Reviewer response for version 1

**DOI:** 10.21956/gatesopenres.14910.r32401

**Published:** 2022-09-12

**Authors:** Bernd Froessler

**Affiliations:** 1 Department of Anaesthesia, Lyell McEwin Hospital, Adelaide, SA, Australia; 2 Discipline of Acute Care Medicine, University of Adelaide, University of Adelaide, SA, Australia

This is an interesting study and provided this reviewer with a lot of learnings. Objectives, outcomes, and conclusions are clearly stated and supported.

 Comments:

**Anaemia in Malawi:**

- Could the authors add the classification for mild, moderate and severe anaemia?

**FCM:**

- From the product information:
  “FERINJECT may be administered by intravenous infusion up to a maximum single dose of 1,000 mg iron (up to a **maximum of 20 mg iron**/kg body weight). Do not administer more than 1,000 mg iron per week.”
- Please modify.

**REVAMP:**

- Are the authors aware that there is another study called REVAMP? https://pubmed.ncbi.nlm.nih.gov/31443707/

**Participants:**

- How many were approached in total and what was the proportion of approached participants who did not agree to be interviewed?
- As many of the misconceptions/fears are around research why did the authors focus only on IV iron and did not conduct participant interviews with the control arm?

**Discussion:**

**Strengths and limitations of the study:**

- The authors could highlight the small sample size.
- Please consider modifying the following sentence and insert “iron deficiency":
  "...IV iron infusion for treating **iron deficiency** anaemia in pregnancy."

Is the work clearly and accurately presented and does it cite the current literature?

Yes

If applicable, is the statistical analysis and its interpretation appropriate?

Yes

Are all the source data underlying the results available to ensure full reproducibility?

Yes

Is the study design appropriate and is the work technically sound?

Yes

Are the conclusions drawn adequately supported by the results?

Yes

Are sufficient details of methods and analysis provided to allow replication by others?

Yes

Reviewer Expertise:

peri-operative and peri-partum patient blood management.

I confirm that I have read this submission and believe that I have an appropriate level of expertise to confirm that it is of an acceptable scientific standard, however I have significant reservations, as outlined above.

# Response

**Published:** 2022-11-04

**Authors:** Lucinda Manda-Taylor

**Affiliations:** Kamuzu University of Health Sciences, Malawi

Thank you very much, Professor Froessler, for your positive and encouraging feedback. The queries and the suggestions you also provided were beneficial. Herewith is my response to the questions you raise and the requests you make.

**"Anaemia in Malawi:**

 Could the authors add the classification for mild, moderate and severe anaemia?"

 Thank you for the advice. We have added the classification and included the following statement “Anaemia status and severity are defined based on the WHO criteria for different haemoglobin cut-offs pregnant women. The ranges for mild to moderate anaemia are classified as 100-109g/l, 70-99g/l and any range lower than 70g/l is classified as severe anaemia”.

 The reference is provided in intext and at the end of the manuscript.

 WHO. Haemoglobin concentrations for the diagnosis of anaemia and assessment of severity. Vitamin and Mineral Nutrition Information System. Geneva, World Health Organization, 2011 (WHO/NMH/NHD/MNM/11.1) ( http://www.who.int/vmnis/indicators/haemoglobin.pdf, accessed 13 September 2022). 

**"FCM:**

 From the product information:

 “FERINJECT may be administered by intravenous infusion up to a maximum single dose of 1,000 mg iron (up to a **maximum of 20 mg iron**/kg body weight). Do not administer more than 1,000 mg iron per week.”

 Please modify."

 Thank you, we have modified it according to your advice and the product insert information.

 "Are the authors aware that there is another study called REVAMP? https://pubmed.ncbi.nlm.nih.gov/31443707/".

 No, we were not aware, and thank you for making us aware of the other study with the same acronym. While sharing the same acronym, I note that that study differs from this one. The study you refer to focuses on preeclampsia as a cause of maternal, fetal and neonatal morbidity and mortality. Our study focuses on anaemia as a cause of maternal, foetal, and neonatal morbidity and mortality. The intervention of interest is IV iron treatment to help improve poor maternal, foetal, and neonatal outcomes. You can find the protocol on trial published in BMJ Open by Mwangi MN, Mzembe G, Moya E *, et al* *.*

 Protocol for a multicentre, parallel-group, open-label randomised controlled trial comparing ferric carboxymaltose with the standard of care in anaemic Malawian pregnant women: the REVAMP trial

*BMJ Open *2021; **11:**e053288. doi: 10.1136/bmjopen-2021-053288

**"Participants:**

 How many were approached in total, and what proportion of approached participants disagreed with being interviewed?"

 We aimed to interview 12-15 pregnant women at each participating site and conduct at least 2 Focus Group Discussions (FGDs) with pregnant women and 6-10 IDIs with healthcare workers. The table below displays our original sample size estimates for the formative study.

**Study Participant sample size estimates**

**Site: Limbe Health Centre, Blantyre**

 No. HCP IDIs (n = 6-10)

 No of PW IDIs (n = 12-15)

 No. of PW FGDs (n = 1)

**Zomba Central Hospital (ZCH)**

 No. HCP IDIs (n = 6-10)

 No of PW IDIs (n = 12-15)

 No. of PW FGDs (n = 1)

**Grand Total**

 No. of HCP IDIs n = 20

 No. PW IDIs n = 30

 No. PW FGDs n = 2

 However, we only managed to conduct a total of 15 in-depth interviews (n=9 in Blantyre (BT) and n=6 in Zomba (ZA) districts) and 2 FGDs (n=6 and n=8) with pregnant women, one in each district (Blantyre and Zomba) because by the time we started conducting interviews in Blantyre, the trial was struggling to recruit pregnant women mainly because of the fears and misconceptions highlighted in the manuscript. The site in Blantyre had to close because of poor recruitment. We conducted only six interviews in Zomba because very few were willing to participate in the qualitative study. The healthcare provider interviews also ran into similar challenges. Trial staff in Blantyre had to be laid off when the site closed, so we could only interview (n=2) trial staff in Blantyre and (n=5) trial staff in Zomba.

 We did not ask the women why they were unwilling to participate in our interviews because we do not feel it is our right to ask someone why when they decline. In sum, the data we collected through IDIs and FGDs, we determined it was sufficient because we were not learning anything new.

 "As many misconceptions/fears are around research, why did the authors focus only on IV iron and did not conduct participant interviews with the control arm?"

 As this was an exploratory study, with an emphasis placed on quickly identifying any concerns participants had with the intervention, we focused on the direct beneficiaries of the intervention and not those in the control arm receiving oral iron tablets because evidence already exists on the safety and efficacy of oral iron tablets.  

**"Discussion:**

**Strengths and limitations of the study:**

 The authors could highlight the small sample size."

 Although the sample size limits the generalisability of our conclusions, the participant’s perceptions, opinions and experiences nonetheless offer insight into the conditions that could support or affect the acceptability of IV iron infusion for treating iron deficiency anaemia in pregnancy.

 Thank you for the advice. We had included this as a limitation.

 "Please consider modifying the following sentence and insert “iron deficiency":

 "...IV iron infusion for treating **iron deficiency** anaemia in pregnancy.""

 Thank you for the suggestions. We have modified the sentence as recommended.

 We hope that our responses help clarify concerns you may have had and that the suggestions have improved the paper.

 Yours sincerely,

 Lucinda Manda-Taylor
